# A novel Zika virus mouse model reveals strain specific differences in virus pathogenesis and host inflammatory immune responses

**DOI:** 10.1371/journal.ppat.1006258

**Published:** 2017-03-09

**Authors:** Shashank Tripathi, Vinod R. M. T. Balasubramaniam, Julia A. Brown, Ignacio Mena, Alesha Grant, Susana V. Bardina, Kevin Maringer, Megan C. Schwarz, Ana M. Maestre, Marion Sourisseau, Randy A. Albrecht, Florian Krammer, Matthew J. Evans, Ana Fernandez-Sesma, Jean K. Lim, Adolfo García-Sastre

**Affiliations:** 1 Department of Microbiology, Icahn School of Medicine at Mount Sinai, New York, New York, United States of America; 2 Global Health and Emerging Pathogens Institute, Icahn School of Medicine at Mount Sinai, New York, New York, United States of America; 3 The Graduate School of Biological Sciences at the Icahn School of Medicine at Mount Sinai, Microbiology Training Area, New York, New York, United States of America; 4 Department of Medicine, Division of Infectious Diseases, Icahn School of Medicine at Mount Sinai, New York, New York, United States of America; NIH, UNITED STATES

## Abstract

Zika virus (ZIKV) is a mosquito borne flavivirus, which was a neglected tropical pathogen until it emerged and spread across the Pacific Area and the Americas, causing large human outbreaks associated with fetal abnormalities and neurological disease in adults. The factors that contributed to the emergence, spread and change in pathogenesis of ZIKV are not understood. We previously reported that ZIKV evades cellular antiviral responses by targeting STAT2 for degradation in human cells. In this study, we demonstrate that *Stat2*^*-/-*^ mice are highly susceptible to ZIKV infection, recapitulate virus spread to the central nervous system (CNS), gonads and other visceral organs, and display neurological symptoms. Further, we exploit this model to compare ZIKV pathogenesis caused by a panel of ZIKV strains of a range of spatiotemporal history of isolation and representing African and Asian lineages. We observed that African ZIKV strains induce short episodes of severe neurological symptoms followed by lethality. In comparison, Asian strains manifest prolonged signs of neuronal malfunctions, occasionally causing death of the *Stat2*^*-/-*^ mice. African ZIKV strains induced higher levels of inflammatory cytokines and markers associated with cellular infiltration in the infected brain in mice, which may explain exacerbated pathogenesis in comparison to those of the Asian lineage. Interestingly, viral RNA levels in different organs did not correlate with the pathogenicity of the different strains. Taken together, we have established a new murine model that supports ZIKV infection and demonstrate its utility in highlighting intrinsic differences in the inflammatory response induced by different ZIKV strains leading to severity of disease. This study paves the way for the future interrogation of strain-specific changes in the ZIKV genome and their contribution to viral pathogenesis.

## Author summary

Zika virus is the most recent viral pathogen to cause a global public health emergency. It is distinct from other flaviviruses in its ability to cause transplacental infection, fetal abnormalities and vector independent transmission through body fluids in humans. Over the last year, there has been rapid progress in the development of animal models, which can be used to study ZIKV pathogenesis. In this study, we demonstrate for the first time that *Stat2*^-/-^ mice are highly susceptible to ZIKV infection and recapitulate aspects of ZIKV pathogenesis and disease. We use this model to delineate whether strain specific differences in ZIKV pathogenesis exist, using diverse strains representing both African and Asian lineages. We show that African strains in general are more virulent than Asian strains and their pathogenicity associates closely with the degree of inflammatory immune response in the CNS of infected mice, and does not necessarily correlate with viral RNA levels. Thus, we establish *Stat2*^*-/-*^ mice as new model to study ZIKV pathogenesis and use it to characterize inherent differences in the virulence among ZIKV strains. More importantly, we also highlight a potential role of the host inflammatory immune response in mediating differential pathogenesis among ZIKV strains.

## Introduction

The genus *Flavivirus* of the family *Flaviviridae* includes important vector-borne human pathogens such as dengue (DENV), yellow fever (YFV), West Nile (WNV), Japanese encephalitis (JEV), and tick-borne encephalitis viruses (TBEV). Many of these viruses are prevalent in the equatorial region of the globe that harbors the arthropod vectors required for their transmission [1–3]. Zika virus (ZIKV) is a recently emerged member of the flaviviruses which has caused large outbreaks in human populations over the last decade and was recognized as a Public Health Emergency of International Concern (PHEIC) by the World Health Organization (WHO) in February 2016. It is a mosquito borne, enveloped virus with a 10.7 kb positive sense single stranded RNA genome. Like other flaviviruses, the ZIKV genome encodes a polyprotein, which is post-translationally processed by cellular and viral proteases into three structural proteins (Capsid/C; pre-membrane/prM; Envelope/E) and seven non-structural proteins (NS1, NS2A, NS2B, NS3, NS4A, NS4B and NS5). The structural proteins protect the genome, participate in virus entry into and exit from the host cell, and are primary targets of the host antibody mediated immune response. Non-structural proteins are required for viral genome transcription and replication, proteolytic processing of the polyprotein and inhibition of cellular innate immune response [1–3]. ZIKV was first isolated in 1947 from a febrile rhesus macaque in the Ziika forest in Uganda, where it circulated between nonhuman primates and sylvatic *Aedes africanus* mosquitoes [4]. Subsequently, it was sporadically diagnosed in Africa and Southeast Asia as a cause of mild self-limiting febrile disease in humans. In 2007, it caused the first large-scale outbreak in the Yap islands, where more than 7,000 individuals were estimated to be infected [3]. In 2013, ZIKV appeared in French Polynesia, where it infected approximately 28,000 individuals [1–3]. Here for the first time, ZIKV infections were found to be associated with increased incidences of Guillain-Barré syndrome, a debilitating neuronal disease in adults. Also for the first time, ZIKV presence was detected in body fluids other than blood (including semen, saliva, urine), and risk of direct human-to-human transmission was observed. In 2014, ZIKV appeared in Brazil and by late 2015 the virus had spread across South and Central America, the Caribbean and the southern parts of the United States [1–3]. Importantly, during spread of ZIKV pandemic in Brazil, infections during pregnancy were linked to fetal malformations such as spontaneous abortion, stillbirth, hydrocephaly and microcephaly, and placental insufficiency to miscarriage, now collectively known as congenital Zika virus syndrome (CZVS) [5].

Phylogenetic analysis of ZIKV genomes reveals African and Asian as two distinct lineages. It has been postulated that ZIKV originated in East Africa, spread to West Africa and Asia nearly 50~100 years ago, before making its appearance in the Western hemisphere in 2014 [6, 7]. The molecular determinants of ZIKV evolution, spread and disease phenotype have not been established yet. Since the 2015 ZIKV epidemic in Brazil, efforts have been put forth by several research groups to develop new *in vitro*, *ex vivo* and *in vivo* models to study ZIKV infection and pathogenesis [2, 8]. Studies on stem cell derived neural progenitor cells and brain organoid models showed neurotropism and pathogenicity of ZIKV [9]. *Ex vivo* human placental tissue models have shown transplacental infection of ZIKV, its preference for trophoblasts and sensitivity to type III IFN [10, 11]. In the last year there have been extensive efforts to develop new animal models to study ZIKV pathogenesis [8]. At the time of the original ZIKV isolation, Dick *et al* tested ZIKV infection in several animal species including mice, cotton rats, guinea pigs and rhesus macaques, and showed that only neonatal mice were prone to symptomatic ZIKV infections [4, 12]. Later, ZIKV infection and disease symptoms were demonstrated in neonatal mice, rhesus macaque and chicken embryos [8, 13–17]. Similar to dengue viruses, ZIKV does not cause infection and disease in wild type adult mice. To overcome this limitation, scientists have used immunocompromised, neonatal or special mouse strains such as SJL strain to study ZIKV pathogenesis [13, 14, 17–19]. Although mice are significantly different than humans, especially in their placental anatomy and gestation period, scientists have demonstrated their utility in reproducing ZIKV pathogenesis consistent with that in humans [20]. Mouse models have been used to demonstrate ZIKV ability to cause fetal abnormalities, deterioration of gonadal tissue and infection through sexual route [8, 13, 21–28]. Immunocompromised mouse models have been useful in recapitulating the neurotropic nature of ZIKV infection and in demonstrating a critical role of IFN induction and signaling for protection against ZIKV [14, 17, 18].

In an earlier report, we showed that ZIKV targets human STAT2 for degradation, thereby reducing induction of IFN stimulated genes (ISGs) and subsequent antiviral responses in human cells, but not in mouse cells [29]. These findings were corroborated by another study which also concluded proteasomal targeting of STAT2 by ZIKV NS5 protein [30]. Following up on these observations we now demonstrate that ZIKV challenge in *Stat2*^*-/-*^ mice results in ZIKV infection and spread to CNS, gonads and other vital organs, with manifestation of neurological symptoms. Further we have used the *Stat2*^*-/-*^ mouse model to compare the virulence and pathogenesis of a panel of ZIKV strains representing different lineages and spatio-temporal isolation histories.

## Results

### *Stat2*^*-/-*^ mice are susceptible to ZIKV infection and display several key aspects of human ZIKV pathogenesis

**Fig 1:**
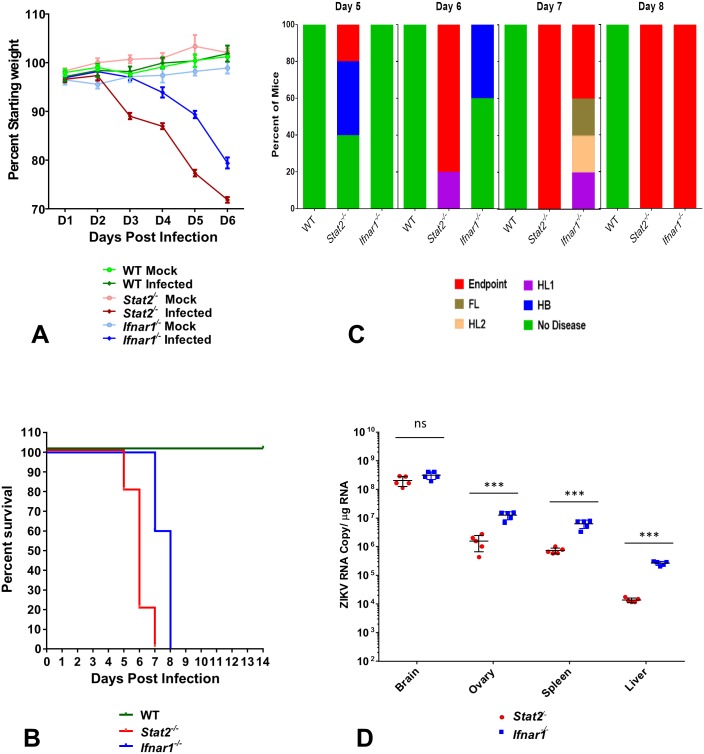
*Stat2*^*-/-*^ mice support ZIKV infection and recapitulate ZIKV pathogenesis and disease. Five to six week old female WT, *Stat2*^*-/-*^ and *Ifnar1*^*-/-*^ C57BL/6 mice (n = 5) were injected with 1,000 PFU of ZIKV strain MR766 by the subcutaneous route in the footpad. (A) Mice were weighed daily and weights are expressed as percentage of body weight prior to infection. Results shown are the mean ± standard error of the mean (SEM). Data are censored at 6 days after infection, as mice in the *Stat2*^*-/-*^ group succumbed to infection. (B) In a parallel set of mice (n = 5) lethality was monitored for 14 days. (C) Clinical signs of ZIKV infection were monitored every day in the group of animals used for survival analysis (n = 5). Clinical signs are abbreviated as HB (hunched back, reduced motility), HL1 (one hind limb paralysis), HL2 (both hind limbs paralyzed), FL (one or both front limbs paralyzed), Endpoint (loss of 25% of initial body weight or dead). The percentage of each group of mice displaying the indicated signs is shown. (D) From the group of mice used to monitor body weight loss (n = 5, day 6 post infection), indicated organs were harvested and ZIKV RNA levels were measured by qRT PCR as described in methods. Error bars represent mean ±standard deviation (SD). Y axis starts at the limit of detection of the assay.

To investigate whether elimination of STAT2 in mice was sufficient to render mice susceptible to ZIKV infection, we challenged *Stat2*^*-/-*^ C57BL/6 mice with Uganda ZIKV (strain MR-766, 1947). We used a subcutaneous route to mimic the natural route of infection and recorded ZIKV replication and pathogenesis. Specifically we injected 1,000 plaque formation units (PFU) of Uganda ZIKV in the footpad of 5–6 week old female *Stat2*^*-/-*^ mice. To compare the sensitivity of these mice to ZIKV with other previously used mouse strains, *Ifnar1*^*-/-*^ and wild type (WT) C57BL/6 mice were also infected using the same protocol [17, 18]. We monitored body weight loss and clinical signs of disease every day. The *Stat2*^*-/-*^ mice started losing weight 3 days post infection, and became morbid, displayed limited movement and succumbed to infection between day 6 to 7 (Fig 1a and 1b). In comparison, *Ifnar1*^*-/-*^ mice showed a delayed onset of disease (Fig 1a and 1b), experienced less weight loss than *Stat2*^*-/-*^ mice, displayed a wider range of neurological symptoms, and succumbed to ZIKV infection between day 7 to 8 post infection (Fig 1a, 1b and 1c). Our results are in line with an earlier report where Uganda ZIKV (MR766) challenge (100 PFU, footpad) resulted in 80% lethality in 5 week old *Ifnar1*^***-/-***^ mice by day 14 [17]. WT mice were refractory to ZIKV infection and did not display any signs of disease (Fig 1a, 1b and 1c). ZIKV RNA measurements on day 6 post infection in the brain, ovary, spleen and liver of *Stat2*^*-/-*^ and *Ifnar1*^*-/-*^ mice showed the highest viral RNA levels in the brain, followed by ovary, spleen and minimum levels in the liver (Fig 1d). Interestingly, despite delayed lethality, the viral RNA levels in *Ifnar1*^*-/-*^ mice were higher than *Stat2*^*-/-*^ mice in all the organs tested, except in the brain. In addition, we also evaluated viremia and viral RNA levels in the brain and ovaries of *Stat2*^*-/-*^ mice on days 2, 4 and 6 post infection (S1 Fig). We observed that Uganda ZIKV reached maximum RNA levels in serum (up to 10^^9^ ZIKV RNA copies/ml) as early as 2 days post infection and showed slightly reduced levels by day 6 (S1a Fig). In comparison ZIKV RNA kept on increasing in brain tissue from day 2 to day 6 post infection (S1b Fig). In the ovaries ZIKV reached maximum copy numbers by day 4 (S1C Fig). These trends of temporal changes in ZIKV RNA in different organs are similar to what has been reported in *Ifnar1*^***-/-***^ mice [17]. Taken together, *Stat2*^*-/-*^ mice support ZIKV infection, with virus spread to CNS, gonads, spleen and liver. In comparison *Ifnar1*^*-/-*^ mice show a delayed onset of disease, but higher viral RNA levels in most of the sample tissues. In addition to STAT2 independent signaling of IFN through the type I IFN receptor, the *Stat2*^*-/-*^ and *Ifnar1*^*-/-*^ differ in their ability to support type I and III IFN signaling [31], which may explain their differential susceptibility to ZIKV induced disease.

### Phylogenetic and amino acid variance analysis of ZIKV strains selected for comparison

**Fig 2:**
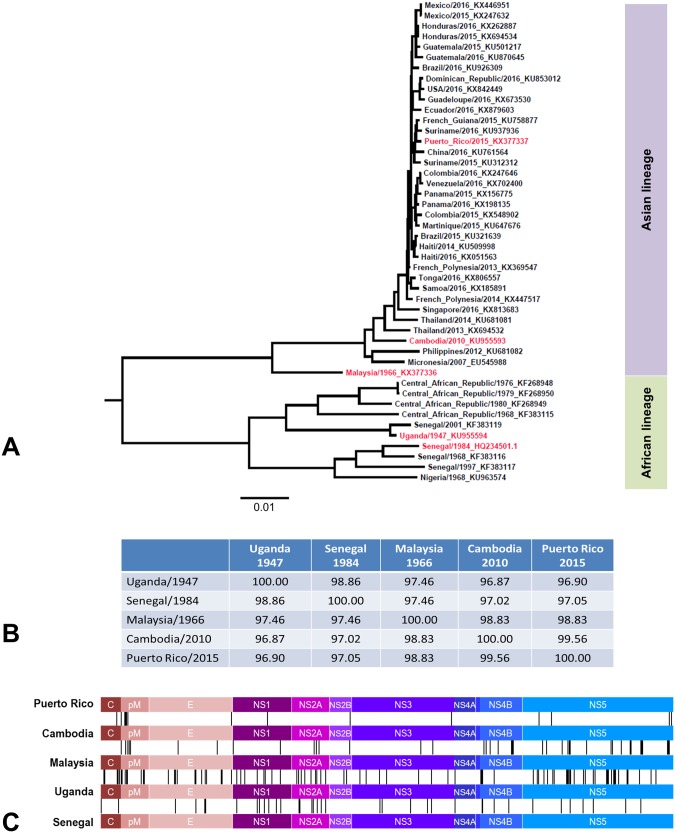
Phylogenetic analysis and amino acid variance of selected ZIKV strains. (A) Neighbor joining phylogenetic tree showing the position of the viral isolates used in this study (in red) in the global ZIKV diversity. (B) Percent amino acid Identity Matrix of the 5 strains used in this study. (C) Graphic representation of the pairwise amino acid changes between the strains used in this study. Each vertical line represents an amino acid difference between the two connected strains. The order of the strains is as in (A). For a detailed description of all the amino acid differences please refer to S1 File.

Phylogenetically, ZIKV can be divided into African and Asian lineages [6, 7]. The African lineage viruses have caused sporadic human infections in the last century, resulting in mild, febrile disease symptoms [7]. The Asian lineage has however emerged at a larger scale displaying vector-borne as well as human-to-human transmission, causing fetal abnormalities and neuronal disease in humans [3]. The factors responsible for emergence and global spread of ZIKV and change in the disease phenotype are not understood presently. Also the impact of genetic changes among ZIKV strains on their pathogenicity has not been studied in detail. To better understand this, we chose a panel of ZIKV strains representing a range of global spatio-temporal isolation. Specifically, we evaluated Uganda 1947 (StrainMR_766), Senegal 1984 (Strain DAKAR 41519) [4, 29, 32], Malaysia 1966 (Strain P6-740), Cambodia 2010 (Strain FSS13025), Puerto Rico 2015 (strain PRVABC59). We refer to these ZIKV strains by the geographical location of their isolation i.e. Uganda, Senegal, Malaysia, Cambodia and Puerto Rico. This panel of ZIKV strains contains ZIKV isolated from 1947 to 2015 from human, mosquito and nonhuman primate hosts and from different locations covering the footprints of the geographical spread of ZIKV (S1 Table). Two of the strains, Uganda and Malaysia, are mouse passaged (S1 Table). A phylogenetic analysis of the complete genome of the selected strains along with other ZIKV genomes reveals their position in the African or Asian lineages (Fig 2a). The list of GenBank accession numbers of the ZIKV strains used to construct the phylogenetic tree is provided in S2 Table. A detailed amino acid sequence comparison of these strains is shown in S1 File. Comparison of the overall amino acid composition shows maximum amino acid differences between the Uganda and the contemporary Puerto Rico strains (Fig 2b). Amino acid differences among the strains are observed throughout the viral polyprotein (Fig 2c), with some regions (such as the amino terminal region of the prM protein, the E, the NS2A and NS5 proteins) showing a higher accumulation of changes, especially between the African and Asian lineages (Fig 2c).

### African ZIKV strains display higher virulence than Asian strains in *Stat2*^*-/-*^ and *Ifnar1*^*-/-*^ mice

**Fig 3:**
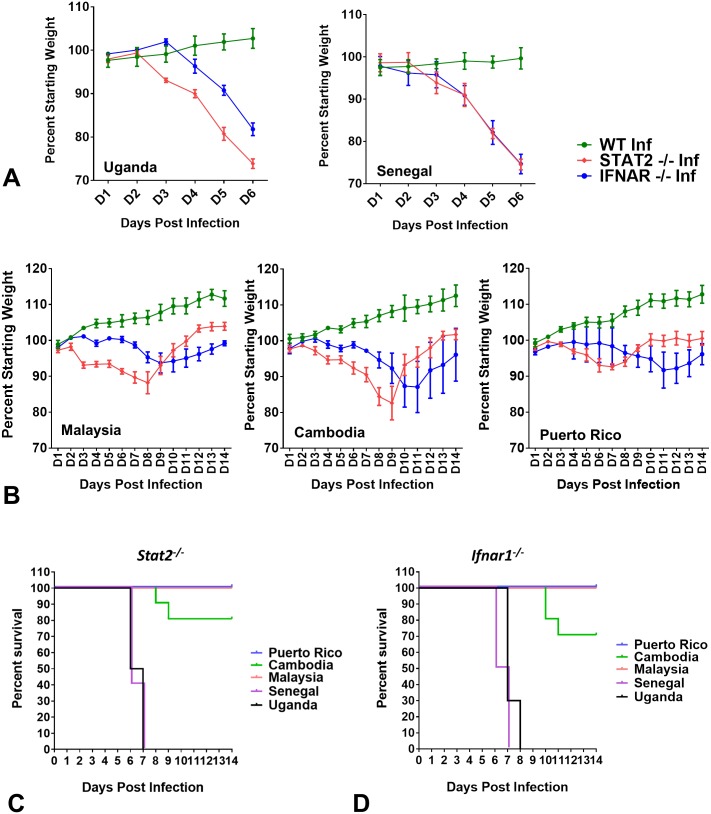
Comparison of weight loss and mortality induced by African and Asian ZIKV strains in mice. Five to six week old male WT, *Stat2*^*-/-*^ and *Ifnar1*^*-/-*^ C57BL/6 mice (n = 5) were injected with 1,000 PFU of the indicated ZIKV strain by the subcutaneous route in the footpad. (A, B) Mice were weighed daily and weights are expressed as percentage of body weight prior to infection. Results shown are the mean ± standard error of the mean (SEM). (A) Data are censored at 6 days after infection, as some mice died. (C, D) In a parallel group, male mice (n = 5) of the indicated genotype were infected with indicated ZIKV strains under similar conditions and data were combined with body weight loss group for survival analysis (total n = 10). ZIKV strains are abbreviated as UG (Uganda), SN (Senegal), ML (Malaysia), CB (Cambodia) and PR (Puerto Rico).

Although there have been several studies showing the utility of different mouse models to study ZIKV pathogenesis, a comprehensive comparative analysis of different ZIKV strains *in vivo* has not been carried out [8]. To address this we set out to compare the pathogenicity of a panel of five ZIKV strains representing African and Asian lineages in *Stat2*^*-/-*^, *Ifnar1*^*-/-*^ and WT mice. Specifically we injected 1,000 PFU of the indicated ZIKV strain into the footpad of male mice of the indicated genotype and monitored them every day for morbidity and mortality. Among the African lineage viruses, the Senegal strain caused similar body weight loss in both *Stat2*^*-/-*^ and *Ifnar1*^*-/-*^ mice (Fig 3a). In comparison Uganda ZIKV caused more weight loss in *Stat2*^*-/-*^ mice than *Ifnar1*^*-/-*^ mice (Fig 3a). In terms of mortality all *Stat2*^*-/-*^ and *Ifnar1*^*-/-*^ mice succumbed to African ZIKV infections between day 6 to 8, with *Stat2*^*-/-*^ mice displaying slightly higher early mortality as compared to *Ifnar1*^*-/-*^ mice (Fig 3c and 3d). Among Asian lineage viruses all strains caused body weight loss, with *Stat2*^*-/-*^ mice showing an earlier onset of morbidity when compared to *Ifnar1*^*-/-*^ mice (Fig 3b). Interestingly when compared to African strains, Asian strains displayed delayed onset of disease and less severe morbidity in both *Stat2*^*-/-*^ and *Ifnar1*^*-/-*^ mice (Fig 3a and 3b). Only the Cambodia strain caused limited mortality at 1,000 PFU in *Stat2*^*-/-*^ (20% mortality) and *Ifnar1*^*-/-*^ mice (30% mortality) (Fig 3c and 3d). All other mice infected with Asian lineage ZIKV strains seemed to recover from disease and regained their original body mass (Fig 3b). In the study by Helen M Lazear *et al*, French Polynesia strain (H/PF/2013) caused 100% lethality in *Ifnar1*^***-/-***^ mice by day 10 at dose of 100 PFUs, which is different from our observations with Asian strains. It could’ve resulted due amino acid differences between different Asian strains. However for African lineage Senegal strain (Dakar 41519) they also observed 100% lethality in 4 week old *Ifnar1*^***-/-***^ mice, which is consistent with our findings. The WT mice did not show any clear symptoms of disease with either Asian or African ZIKV strains. Taken together these data show inherent differences in morbidity and lethality induced by African and Asian ZIKV strains in *Stat2*^*-/-*^ and *Ifnar1*^*-/-*^ mice.

### ZIKV pathogenicity does not directly correlate with the level of viral replication in mice

**Fig 4:**
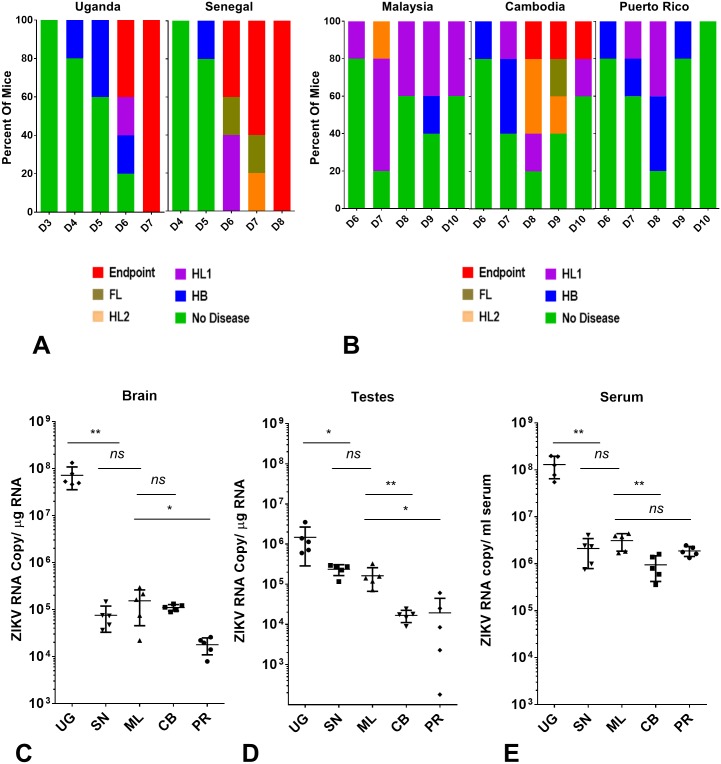
Comparison of neurological disease and viral RNA levels among African and Asian ZIKV strains after infection in *Stat2*^*-/-*^ mice. (A, B) Clinical signs of ZIKV infection were monitored every day in the group of animals used for survival analysis in Fig 3 (n = 5). Clinical signs are abbreviated as HB (hunched back, reduced motility), HL1 (one hind limb paralysis), HL2 (both hind limbs paralyzed), FL (one or both front limbs paralyzed), Endpoint (loss of 25% of initial body weight or dead). The percentage of each group of mice displaying the indicated signs is shown. (A) Data represent clinical signs observed between day 3 to day 7 post infection for the Uganda strain and day 4 to day 8 post infection for the Senegal strain. (B) Data represent clinical signs induced by Asian lineage strains between day 6 to day 10 post infection. (C, D, E). Five to six week old male *Stat2*^*-/-*^ mice (n = 5) were infected with indicated ZIKV as described in Fig 3. On day 6 post infection indicated organs were harvested and ZIKV RNA levels were measured by qRT PCR as described in methods. Error bars represent mean ±standard deviation (SD). Y axis starts at the limit of detection of the assay.

Next we examined clinical symptoms induced by different ZIKV strains in *Stat2*^*-/-*^ mice and measured ZIKV viral RNA levels in the serum, CNS and gonads. Clinical signs of ZIKV disease started with reduced motility and hunched posture followed by partial or complete paralysis in hind limbs, front limbs and lethality. In most cases mice suffering front limb paralysis succumbed to the disease the same or next day. We observed that African lineage strains induced higher mortality and severe neurological symptoms in a short period as compared to Asian lineage strains (Fig 4a). Although all animals infected with African lineage strains succumbed to infection, the Senegal strain induced more severe neurological symptoms such as paralysis in all limbs (Fig 4a). Among Asian lineage strains the Cambodia strain presented more severe symptoms including front limb paralysis and lethality, followed by the Malaysia and Puerto Rico strains (Fig 4b). The Puerto Rico strain was the least pathogenic among all strains and all mice recovered from disease symptoms by day 10 (Fig 4b). Similar trends were observed in *Ifnar1*^*-/-*^ mice as well (S1 Fig). In general Asian lineage strains presented delayed onset but prolonged, although less severe, neurological symptoms (up to 4 days) as compared to African strains. We also measured viral RNA levels in serum, brain and testes of mice infected with different ZIKV strains at day 6 post infection. Interestingly the Uganda strain showed orders of magnitude higher viral RNA level compared to all other strains including Senegal (Fig 4b, 4c and 4d), especially in brain tissue. Similarly, among Asian strains the Malaysia strain showed elevated viral RNA compared to Cambodia and Puerto Rico ZIKV (Fig 4b, 4c and 4d). Both Uganda and Malaysia strains are mouse passaged strains (S1 Table), which may explain their higher replication compared to their lineage specific counterparts. In any case, these data clearly show that pathogenicity manifested by different ZIKV strains is not in direct correlation with the level of viral replication in mice.

### Degree of ZIKV virulence correlates with levels of inflammatory cytokine induction in mice

**Fig 5:**
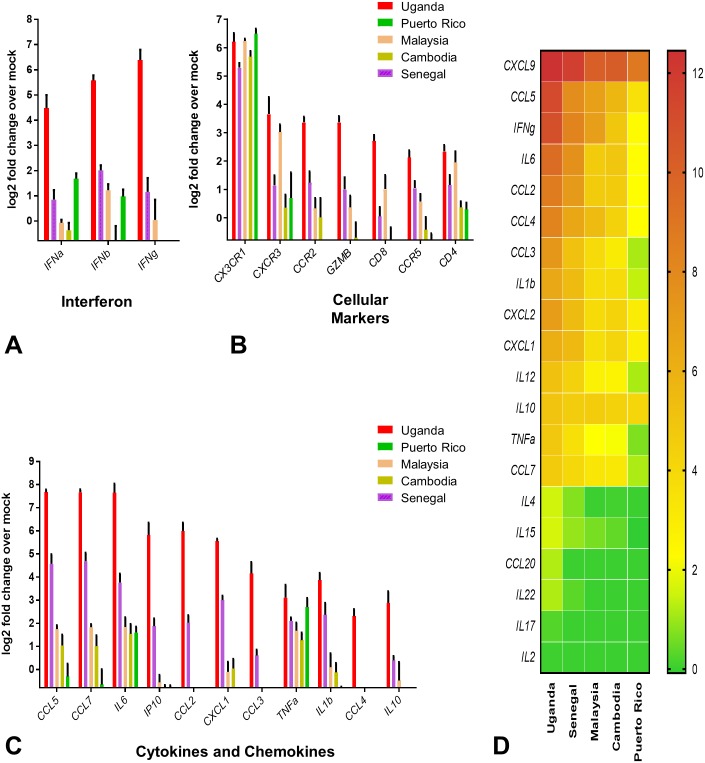
Comparison of inflammatory immune response induced by African and Asian ZIKV strains in the CNS of *Stat2*^*-/-*^ mice. (A, B, C) Brain tissue homogenates were prepared from the group of male *Stat2*^*-/-*^ mice used to measure ZIKV RNA (n = 5) (Fig 4C, 4D and 4E) and a control group of age matched, mock infected male *Stat2*^*-/-*^ mice (n = 5). Gene expression was measured by qRT PCR as described in the methods section. Fold change of the gene expression over the mock was calculated and plotted on the graphs. Data are presented as mean ±standard deviation (SD) of log 2 fold increase over mock controls. Genes are grouped into indicated functional categories. (D) In the same set of brain homogenates, indicated cytokine and chemokine levels were measured by multiplex ELISA assay, as described in the methods section. Data are presented as mean of log 2 fold increase over mock controls.

Earlier reports had shown that ZIKV infection in animal models is associated with the activation of immune responses, cellular infiltration, inflammation and neural damage in the CNS [13, 15, 25]. To test if this may be the underlying cause of the difference in pathogenicity between the African and Asian lineages of ZIKV, we measured markers associated with IFN induction, expression of inflammatory cytokines and recruitment of immune effector cells in the brain of *Stat2*^*-/-*^ mice on day 6 post infection. For measuring cytokine and chemokine induction, we measured both mRNA expression and protein levels by qRT-PCR and multiplex ELISA, respectively. We observed higher levels of type I and type II IFNs and cytokine induction by African lineage strains as compared to the Asian lineage strains (Fig 5a, 5b, 5c and 5d). Specifically, we observed enhanced levels of hallmark inflammatory cytokines such as IL6, IP10, TNFα, IFNγ, as well as chemokines associated with T cell infiltration (*CCL3*, *CCL4*, CCL5, and *CXCL9*) and cytolytic response (*GZMB*, *IFNγ*), monocyte/macrophage infiltration (*CCL2*, *CCL7*), and neutrophil migration (*CXCL1* and *CXCL2*). Additional cytokines with known antiviral activity were also induced, such as *IL-1b* and *IL15* [33, 34]. In general, these cytokines were induced to a greater extent by the African strains than Asian strains (Fig 5a, 5b, 5c and 5d). Markers associated with infiltration of T cells (*CD4*, *CD8*, *CCR5*, *CXCR3*), monocytes (*CCR2*, *CCR5*) and NK cells (*CCR5*) were also differentially upregulated, while microglial activation appeared to be uniformly induced irrespective of the ZIKV strain as measured by *CX*_*3*_*CR1* expression (Fig 5b). Taken together induction of an exacerbated inflammatory immune response in the CNS is a better indicator of ZIKV pathogenicity in mice than viral RNA levels. To test whether gender of the mice affected ZIKV strain specific pathogenesis, we repeated ZIKV challenge with Senegal, Cambodia and Puerto Rico strains in female *Stat2-/-* mice as done before with male mice. We observed similar pattern of body weight loss, ZIKV viral RNA and inflammatory cytokine levels in the mouse brain (S3a, S3b and S3c Fig) as seen in male mice (Figs 3–5), thus differences in African and Asian ZIKV strain specific pathogenesis were independent of mouse gender.

## Discussion

In November 2016 WHO declared that ZIKV is no longer a public health emergency, however it remains a serious public health concern, especially in the light of recent studies showing fetal microcephaly and other long-term cognitive disorders resulting from ZIKV infection [35]. From a scientific perspective, there is still an extensive knowledge gap on ZIKV evolution, biology and pathogenesis. From the first isolation of ZIKV in 1947 until 2015, there were only three studies which tested the virus' pathogenic potential in animal models [12, 13, 36]. Since then there have been several more which have utilized various mouse models to study different aspect of ZIKV pathogenesis and revealed critical evidence of neuropathology and fetal abnormalities associated with ZIKV infection [8]. Mouse models remain an attractive option because of their amenability for genetic manipulation and the availability of a vast repertoire of mouse strains, which allows investigation of the role of specific host genetic loci in viral pathogenesis. Using mouse strains deficient in various components of antiviral signaling, researchers had shown a critical role of type I IFN signaling in preventing ZIKV infection in mice [14, 17, 18]. Shannan L Rossi et al compared susceptibility of A129 (lack type I IFN response) and AG129 (lack type I and II IFN response) mice to ZIKV challenge and found the latter to be more prone to severe disease [19]. This highlighted the role of type I and II IFN response against ZIKV. Later on *irf3*^***-/-***^*irf5*^***-/-***^*irf7*^***-/-***^ triple knock out mice which are compromised in type I, II and III IFN induction were shown to be more susceptible to ZIKV induced disease as compared to *Ifnar1*^***-/-***^ mice (lack type I IFN response) [17], which pointed to importance of type II and III IFN response against ZIKV. In our previous studies on the role of ZIKV NS5 protein in antagonizing the antiviral host response, we found that, similar to DENV, ZIKV targets STAT2 for degradation in human cells and not in mouse cells, although probably through the involvement of different host co-factors [29]. This knowledge led us to hypothesize that STAT2 could be critical for preventing ZIKV infection in animal models. Indeed in our study, *Stat2*^*-/-*^ mice were highly susceptible to ZIKV infection and disease. In comparison *Ifnar1*^*-/-*^ mice showed in general delayed disease symptoms and less body weight loss due to ZIKV infection than *Stat2*^*-/-*^ mice. While *Ifnar1*^*-/-*^ mice lack type I IFN signaling, *Stat2*^*-/-*^ mice lack both type I and type III IFN signaling [31]. This may explains the difference in their susceptibility to ZIKV induced disease and suggests a critical role of type III IFN in mediating antiviral responses against ZIKV. This is in accordance with the findings of Bayer *et al* [10] who showed protective role of type III IFN against ZIKV infection in human placental tissue. However, for the differences observed between *Stat2*^*-/-*^ and *Ifnar1*^***-/-***^ mice, we cannot exclude the possible role of STAT2 independent, IFNAR1 dependent, type I IFN signaling. Immunocompromised mouse models are useful options when WT mice are refractory to viral infection. In our study *Stat2*^*-/-*^ mice recapitulated ZIKV tissue tropism to CNS and gonads and displayed neurological symptoms of various degrees, which are consistent with human disease. It will be interesting to test ZIKV infection of *Stat2*^*-/-*^ as well as *Stat2*^*-/+*^ heterozygous mice in pregnancy models to see whether they can simulate the transplacental infection and neuroteratogenic properties of ZIKV.

In *vitro* stem cell derived models have been very useful in confirming the neurotropic nature and teratogenic potential of ZIKV [9, 37–39]. In recent studies of gene expression profiles induced by African and Asian ZIKV in neural progenitor cells, it was found that African ZIKV induces higher levels of transcripts associated with inflammation and cell death which is consistent with our findings in infected *Stat2*^-/-^ mice [39, 40]. However an independent study using human induced pluripotent stem cell (iPSC) derived brain organoids showed that African and Asian lineage ZIKV strains display similar inhibitory effects on neuronal differentiation and organoid development [37]. In our study, African and Asian strains display drastically different virulence in mice. We found that African strains tend to be more lethal, with severe short episodes of neural malfunction, whereas Asian strains yield wider spectrum of neurological symptoms which last for longer period and seldom result in mortality. Interestingly the ZIKV RNA levels in CNS or other organs did not directly correlate with virulence. Among African strains, Uganda ZIKV replicated to higher levels than the Senegal strain although they manifested similar virulence. Similarly among Asian strains Malaysia replicated to slightly higher levels than the Cambodia strain, however Cambodia ZIKV displays more severe disease as compared to Malaysia ZIKV. Both Uganda and Malaysia strains are mouse passaged, which may explain higher replication levels as compared to their lineage specific counterparts.

Consistent with earlier reports showing CNS inflammation in ZIKV infected neonatal mice and Rhesus macaques [15, 25], infection of *Stat2*^*-/-*^ mice with all ZIKV strains led to inflammatory immune responses in the brain. Importantly, the differences in ZIKV strain-specific pathogenesis were found to be closely correlated with the level of induction of inflammatory cytokines and possible recruitment of immune effector cells to the CNS of infected mice. These findings highlight that disease severity induced by ZIKV infection is more likely a product of the host response, rather than the level of viral replication, at least in immunocompromised mouse models. We also observed that, other than Uganda, all ZIKV strains achieved similar viral RNA levels in the mouse brain, however this led to variable induction of IFNα, IFNβ, and IFNγ. This could have resulted from differences in the ability of ZIKV strains to antagonize the induction of IFN. As these mice lacked STAT2, the NS5 mediated IFN antagonism through degradation of STAT2 does not account for these differences. For ZIKV and other flaviviruses, IFN antagonism has also been attributed non-structural proteins other than NS5 [30, 41]. It would be interesting to investigate whether amino acid changes in specific viral proteins among different ZIKV strains result in differential IFN antagonism in mouse and in human systems.

It is critical to understand the factors which facilitated sudden emergence and spread of ZIKV, to take preventive measures in the future. It is likely that global warming, deforestation, increased prevalence of *Aedes sp*. mosquitoes, increased global transportation of ZIKV infected individuals and immunological naivety of human populations in new areas may have facilitated these events. However it is also plausible that during the course of its evolution ZIKV acquired changes in its genome [6] that may have driven its global spread. In humans, ZIKV induced neurological symptoms and fetal abnormalities have been only reported in the case of Asian strains [3]. Interestingly, in our study Asian strains displayed prolonged neurological malfunction in mice as compared to the African strains. However it is important to acknowledge that human sero-epidemiological data of African ZIKV is limited and the true phenotype of disease in ZIKV endemic areas in Africa needs to be investigated in detail.

ZIKV is not the first arbovirus to show sudden emergence and global spread. In fact, ZIKV has followed a trajectory displayed earlier by chikungunya virus, which in the course of evolution acquired a small number of mutations allowing it to use additional species of *Aedes* mosquitos for transmission [42]. Interestingly from the time of ZIKV discovery to its emergence and spread, several changes have been found spread across its genome [6, 7]. These changes may affect the rate of ZIKV replication, expand its host range, alter the ZIKV tissue tropism, and/or enhance the ability of ZIKV to induce, antagonize or escape the host immune response. Our studies show ZIKV strain specific differences in inducing disease in mice, which are associated with differential activation of proinflammatory cytokines, emphasizing the possibility that different ZIKV strains might also display phenotypic differences in humans. If so, these differences might have contributed to the ZIKV global spread and to changes in the spectrum of disease in humans during the recent years. It will be critical to employ reverse genetics tools to systematically address the role of specific genetic changes in ZIKV strains in their pathogenesis and host range. The *Stat2*^*-/-*^ mouse model described here could be a useful tool to evaluate the pathogenesis of recombinant ZIKV carrying different combinations of these genetic changes.

## Materials and methods

### ZIKV strains and mouse genotypes

The following ZIKV strains were used in this study: Uganda 1947 (Strain MR 766; GenBank HQ234498.1), Puerto Rico 2015 (Strain PRVABC59; GenBank KU501215.1), Cambodia 2010 (Strain FSS13025; GenBank: JN860885.1), Malaysia 1966 (Strain P6-740; GenBank HQ234499.1) and Senegal 1984 (Strain DAKAR 41519; GenBank HQ234501). The Uganda strain was obtained from American type culture collection (ATCC). All virus stocks were grown in Vero cells and quantified by plaque assay. A summary of isolation history of all ZIKV strains and related references is provided in S1 Table. Malaysia, Cambodia and Senegal ZIKV strains were obtained from Dr. Robert B. Tesh, The World Reference Center for Emerging Viruses and Arboviruses (“WRCEVA”), through the University of Texas Medical Branch at Galveston. Puerto Rico ZIKV strain was provided by Barbara W. Johnson. *Stat2*^*-/-*^ C57BL/6 mice were kindly provided by Dr. Christian Schindler. *Ifnar1*^*-/-*^ C57BL/6 mice deficient in IFN α/β receptor -/- were kindly provided by Dr. Thomas Moran. WT C57BL/6 mice were obtained from Jackson Laboratories.

### Virus stock growth and quantification

Virus stocks were grown in Vero (African green monkey kidney) cells and in some cases passaged in C6/36 (*Aedes albopictus* cells), which were obtained from ATCC. Virus titers of the stocks were measured by plaque formation assay on Vero cells. Specifically, 100 μl of 10 fold virus stock serial dilution was incubated for 2 hour on Vero cell monolayer in 12 well plate format. Finally cells were overlaid with 1 ml DMEM (Invitrogen) supplemented with 0.8% methyl cellulose, 2% FBS, and 50 μg/ml penicillin and streptomycin. Cells were incubated for 5 days at 37°C, fixed with 4% PFA, and stained with crystal violet for plaque visualization.

### ZIKV infection and clinical evaluation of mice

Prior to ZIKV inoculation, mice were anesthetized by intraperitoneal injection of a mixture of ketamine (100 μg per gram of body weight) and xylazine (5 μg per gram). Afterwards, 1,000 PFUs of the indicated ZIKV strain were injected subcutaneously in the mouse footpad in 25 μl of phosphate-buffered saline (PBS). Mice were monitored daily for weight loss and clinical signs. Examination included appearance, posture, motility, hind and front limb paralysis. Animals that showed more than 25% weight loss or complete paralysis were humanely euthanized.

### ZIKV quantification by qPCR

Infected mice were euthanized by CO_2_ asphyxiation and organs were harvested aseptically. Tissue homogenates were prepared in PBS supplemented with 0.3% BSA, using metal beads and a FastPrep24 system (MP Biomedicals). ZIKV RNA quantification was conducted as previously described. Briefly, total RNA was isolated from 200 μl tissue homogenate using RNeasy tissue Kit (Qiagen) or 50 μl plasma using QIAamp viral RNA kit (Qiagen). In total, 10ng of RNA or 5 μl of plasma RNA was used with ZIKV-specific primers (5′-TTGGTCATGATACTGCTGATTGC-3′ and 5′-CCYTCCACRAAGTCYCTATTGC-3′) and probe (5′-6FAM-CGGCATACAGYATCAGGTGCATWGGAG-MGBNFQ-3′) (ThermoFisher) and the LightCycler 480 Master Hydrolysis Probes kit (Roche Applied Science, Indianapolis, MD) using the LightCycler 480 II Real Time PCR System (Roche Applied Science). Sequence of the primers and probe targeting ZIKV have been modified from previously published sequences [43] and designed against genomic regions conserved in different ZIKV strains used in the study. RNA quantification was achieved by fitting to an *in vitro* transcribed RNA standard.

### Gene expression quantification by qRT-PCR

Total RNA was isolated from cells using Trizol reagent (Invitrogen). Reverse transcription was performed with the high-capacity cDNA reverse transcription kit (Applied Biosystems). qPCR was done in duplicates using SYBR green I master mix (Roche) in a Roche Light Cycler 480. Relative mRNA values were calculated using ΔΔCt method with 18s RNA as an internal control, and shown as fold change by normalizing to mock control. The sequences of primers used in the study are listed in S3 Table.

### Cytokine and chemokine protein quantification

Protein levels of cytokines/chemokines in clarified tissue homogenates were evaluated using a multiplex bead array assay. All antibodies and cytokine standards were purchased as antibody pairs from R&D Systems or PeproTech. Individual magnetic bead regions (Luminex) were coupled to cytokine-specific capture antibodies according to the manufacturer’s recommendations and as previously described [44]. Conjugated beads were washed and kept at 4°C until use. Biotinylated polyclonal antibodies were used at twice the concentrations recommended for a classical ELISA according to the manufacturer. All assay procedures were performed in assay buffer containing PBS supplemented with 1% normal mouse serum (Invitrogen), 1% normal goat serum (Invitrogen), and 20 mM Tris-HCl (pH 7.4). The assays were performed using 1,500 beads per set of each of cytokines measured per well in a total volume of 50 μl. The plates were read on a Luminex MAGPIX platform. For each bead set, >50 beads were collected. The median fluorescence intensity of these beads was recorded and used for analysis with the Milliplex software using a 5P regression algorithm.

### Phylogenetic analysis

To prepare a phylogenetic tree presenting the global diversity of the Zika virus strains, we downloaded all the available complete genomes from the Virus Pathogen Resource database (www.viprbrc.org/). From these genomes we manually selected a subset of sequences representative of the complete diversity to build the phylogenetic tree. The final selection contained one viral genome from each country and year available, including the 5 strains used in this study. Viral sequences were aligned using the MUSCLE algorithm in MEGA7 (version 7.0.20) and a neighbor joining tree was built using the program Geneious (version 9.1.5). The tree was visualized with the program FigTree and rooted at the midpoint.

### Statistical analysis

All data were analyzed with GraphPad Prism software (Graphpad Software, Inc). For survival analysis, Kaplan-Meier survival curves were analyzed by the log-rank test. An unpaired Student's t-test was used to determine significant differences in viral RNA levels. Values were considered statistically significant when *p<0.05, **p<0.01, ***p<0.001, and ****p<0.0001, ^*ns*^p>o.o5 (not significant). Data are given as mean ± SD or mean ± SEM as indicated; ‘n’ refers to the sample size.

### Ethics statement

This study was carried out in strict accordance with recommendations in the Guide for the Care and Use of Laboratory Animals of the National Institutes of Health. All mouse procedures were approved by Institutional Animal Care and Use Committee (IACUC) of the Icahn School of Medicine at Mount Sinai and performed in accordance with the IACUC guidelines (Protocol # IACUC-2016-0026: “Developing animal models of flavivirus pathogenesis”).

## Supporting information

S1 Fig Kinetic analysis of ZIKV replication in *Stat2*^*-/-*^ mice. Three groups of five to six week old female *Stat2*^*-/-*^ B6 mice (n = 5) were injected with 1,000 PFU of ZIKV strain MR 766 by the subcutaneous route in the footpad. Indicated organs were harvested on indicated days (D2 = 2 days post infection, D4 = 4 days post infection, D6 = 6 days post infection) and ZIKV RNA levels were measured by qRT PCR as described in methods. D6 group is the same set of animals used to measure ZIKV RNA in Fig 1D. Error bars represent mean ±standard deviation (SD). Y axis starts at the limit of detection of the assay.
(TIF)

S2 Fig Comparison of clinical signs induced by African and Asian ZIKV strains in *Ifnar1*^*-/-*^ mice. (A, B) Clinical signs of ZIKV infection were monitored every day in the group of animals used for survival analysis in Fig 3B (n = 5). Clinical signs are abbreviated as HB (hunched back, reduced motility), HL1 (one hind limb paralysis), HL2 (both hind limbs paralyzed), FL (one or both front limbs paralyzed), Endpoint (loss of 25% of initial body weight or dead). The percentage of each group of mice displaying the indicated signs is shown. (A) Data represent mice infected with African lineage strains observed between day 3 to day 7 post infection for Uganda and day 4 to day 8 post infection for Senegal strains. (B) Data represent Asian lineage strains between day 8 to day 12 post infection.
(TIF)

S3 Fig Comparison of ZIKV pathogenesis in female *Stat2*^*-/-*^ mice. (A) Six week old female *Stat2*^*-/-*^ C57BL/6 mice (n = 3 for mock; n = 4 other groups) were injected with 1,000 PFU of the indicated ZIKV strain by the subcutaneous route in the footpad. Mice were weighed daily and weights are expressed as percentage of body weight prior to infection. Results shown are the mean ± SEM. Data are censored at 6 days after infection, as mice were euthanized for brain harvesting. (B) ZIKV RNA levels were measured in the brain homogenates by qRT PCR as described in methods. Error bars represent mean ±standard deviation (SD). ZIKV strains are abbreviated as SN (Senegal), CB (Cambodia) and PR (Puerto Rico). (C) Mouse cytokine mRNA expression in brain was measured by qRT PCR as described in the methods section. Fold change of the gene expression over the mock was calculated and plotted on the graphs. Data are presented as log 2 fold increase over mock controls mean ±standard deviation (SD).
(TIF)

S1 File Amino acid sequence alignment of the ZIKV strains used in the study. (DOCX)

S1 Table Source host, isolation and passage history of ZIKV strains used in the study. AP61 = *Aedes pseudoscutellaris* cells; BHK = baby hamster kidney epithelial cells; C6/36 = *Aedes albopictus* cells; SM = suckling mouse; Vero = African green monkey kidney cells.
(XLSX)

S2 Table List of GenBank accession numbers of ZIKV reference sequences used for phylogenetic analysis. (DOCX)

S3 Table Sequences of qRT PCR primers used in the study. (XLSX)
