# Supplementary figures and images for: A novel Zika virus mouse model reveals strain specific differences in virus pathogenesis and host inflammatory immune responses
Source: PLoS Pathog. 2017 Mar 9;13(3):e1006258. doi: 10.1371/journal.ppat.1006258 (PMC5373643; doi:10.1371/journal.ppat.1006258)

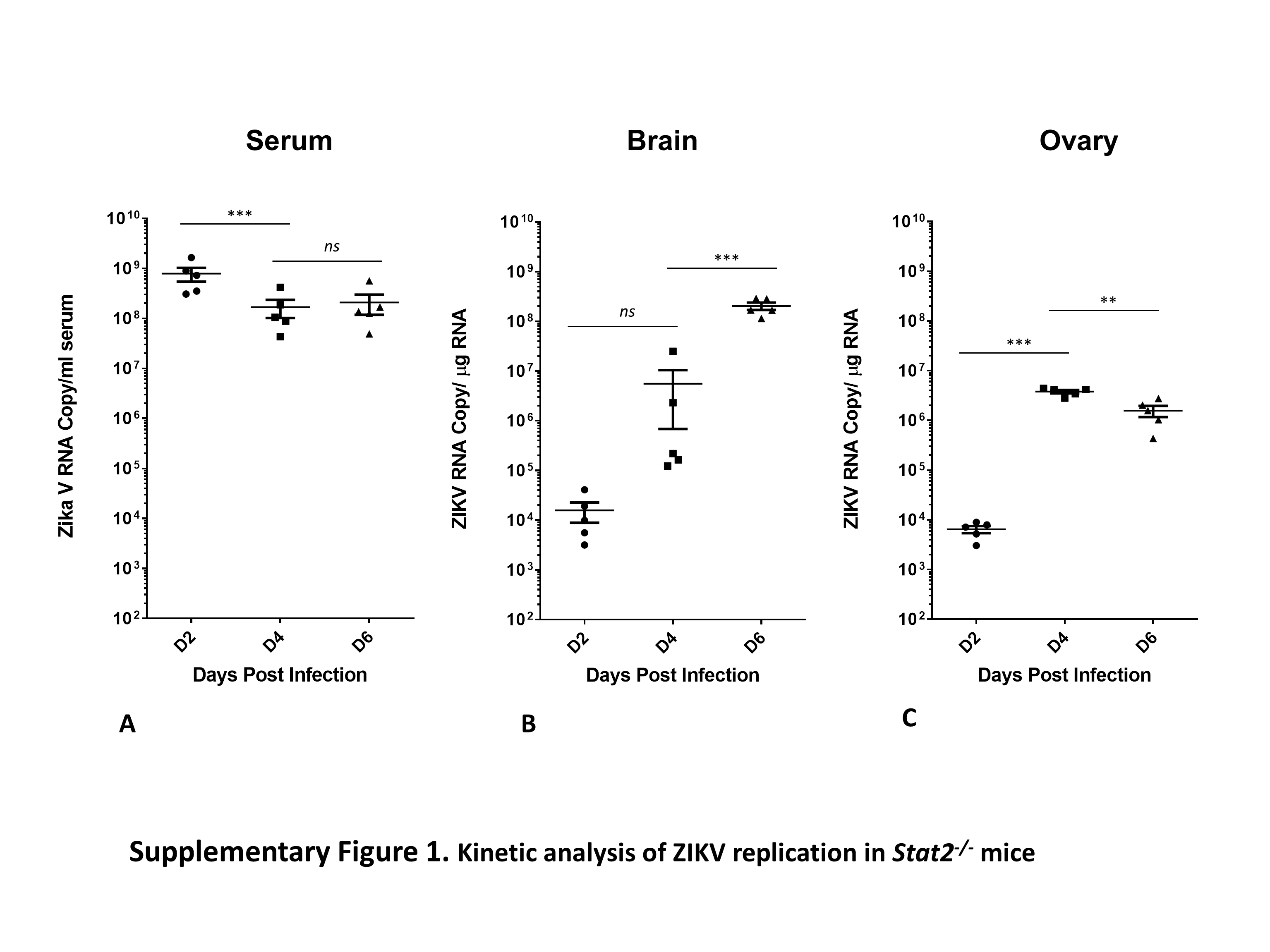

Supplement: S1 Fig — Three groups of five to six week old female Stat2-/- B6 mice (n = 5) were injected with 1,000 PFU of ZIKV strain MR 766 by the subcutaneous route in the footpad. Indicated organs were harvested on indicated days (D2 = 2 days post infection, D4 = 4 days post infection, D6 = 6 days post infection) and ZIKV RNA levels were measured by qRT PCR as described in methods. D6 group is the same set of animals used to measure ZIKV RNA in Fig 1D. Error bars represent mean ±standard deviation (SD). Y axis starts at the limit of detection of the assay. (TIF) [file ppat.1006258.s001.TIF]

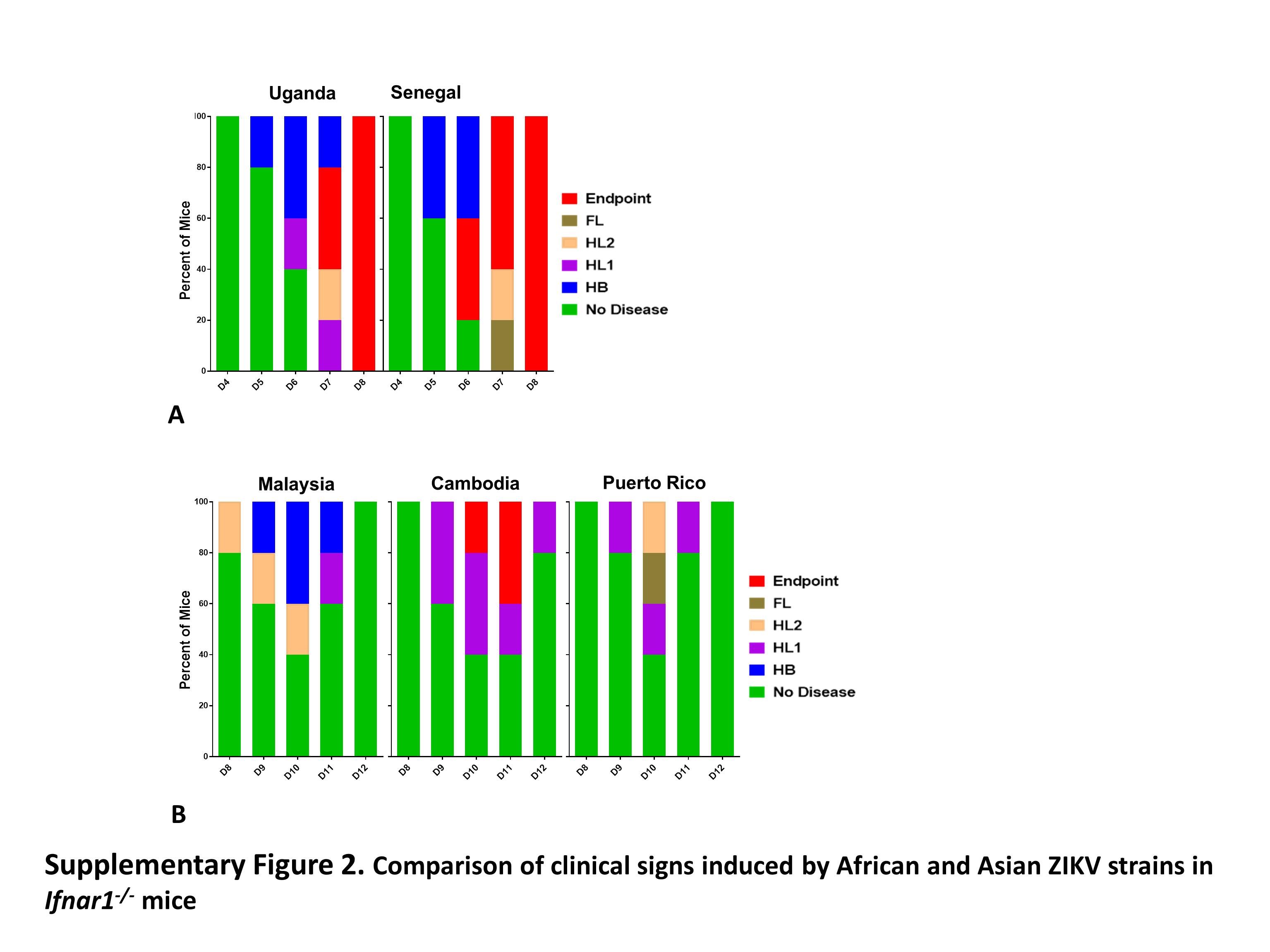

Supplement: S2 Fig — (A, B) Clinical signs of ZIKV infection were monitored every day in the group of animals used for survival analysis in Fig 3B (n = 5). Clinical signs are abbreviated as HB (hunched back, reduced motility), HL1 (one hind limb paralysis), HL2 (both hind limbs paralyzed), FL (one or both front limbs paralyzed), Endpoint (loss of 25% of initial body weight or dead). The percentage of each group of mice displaying the indicated signs is shown. (A) Data represent mice infected with African lineage strains observed between day 3 to day 7 post infection for Uganda and day 4 to day 8 post infection for Senegal strains. (B) Data represent Asian lineage strains between day 8 to day 12 post infection. (TIF) [file ppat.1006258.s002.TIF]

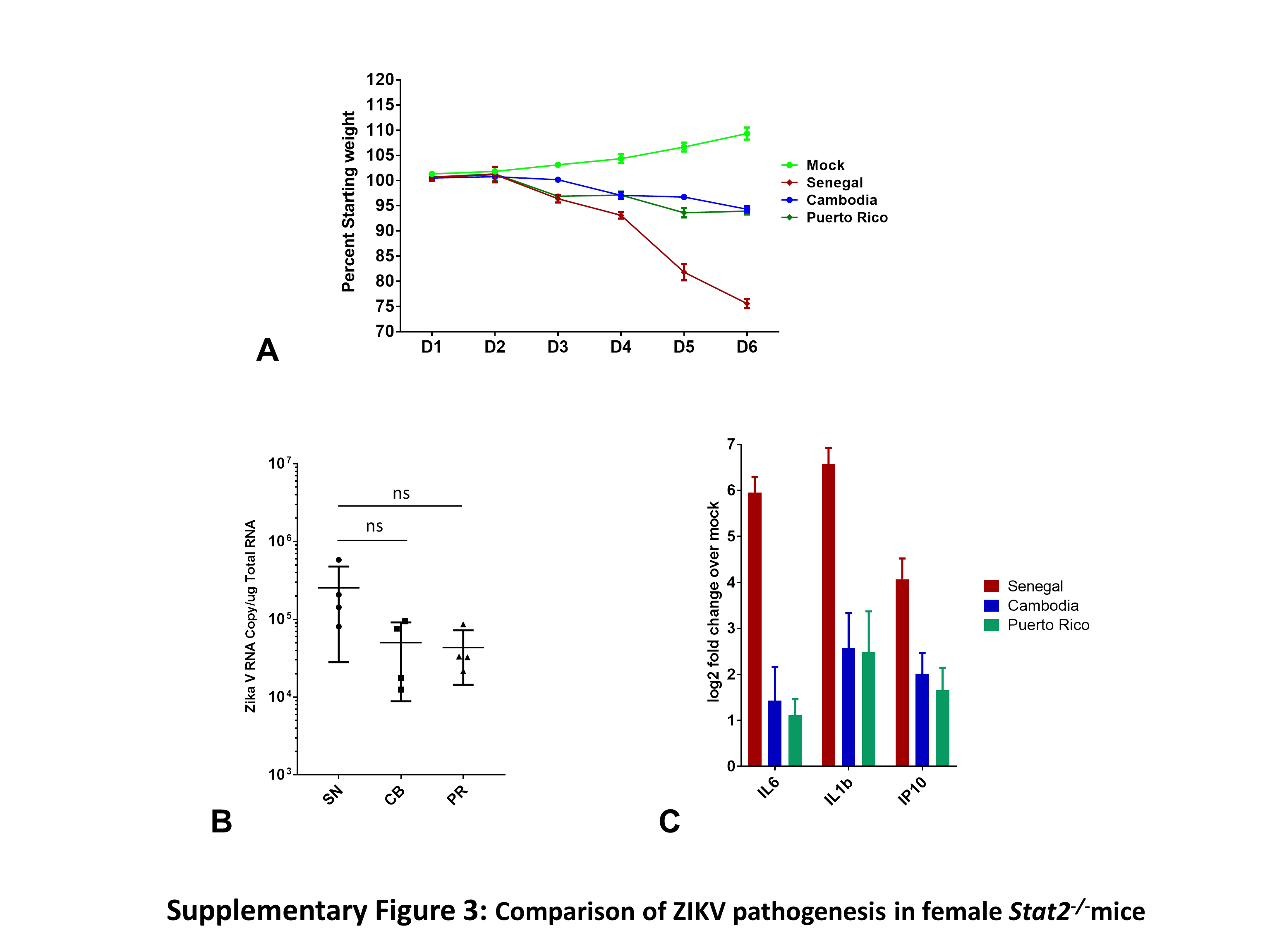

Supplement: S3 Fig — (A) Six week old female Stat2-/- C57BL/6 mice (n = 3 for mock; n = 4 other groups) were injected with 1,000 PFU of the indicated ZIKV strain by the subcutaneous route in the footpad. Mice were weighed daily and weights are expressed as percentage of body weight prior to infection. Results shown are the mean ± SEM. Data are censored at 6 days after infection, as mice were euthanized for brain harvesting. (B) ZIKV RNA levels were measured in the brain homogenates by qRT PCR as described in methods. Error bars represent mean ±standard deviation (SD). ZIKV strains are abbreviated as SN (Senegal), CB (Cambodia) and PR (Puerto Rico). (C) Mouse cytokine mRNA expression in brain was measured by qRT PCR as described in the methods section. Fold change of the gene expression over the mock was calculated and plotted on the graphs. Data are presented as log 2 fold increase over mock controls mean ±standard deviation (SD). (TIF) [file ppat.1006258.s003.TIF]
