# Supplementary material for: A novel Zika virus mouse model reveals strain specific differences in virus pathogenesis and host inflammatory immune responses
Source: PLoS Pathog. 2017 Mar 9;13(3):e1006258. doi: 10.1371/journal.ppat.1006258 (PMC5373643; doi:10.1371/journal.ppat.1006258)
Supplement: S1 File — (DOCX) [file ppat.1006258.s004.docx]

Uganda/1947 MKNPKKKSGGFRIVNMLKRGVARVNPLGGLKRLPAGLLLGHGPIRMVLAILAFLRFTAIK 60

**Capsid**

Senegal/1984 ·····R······················································ 60

Malaysia/1966 ························S·F································· 60

Cambodia/2010 ························S·F································· 60

Puerto_Rico/2015 ························S·F································· 60

Uganda/1947 PSLGLINRWGSVGKKEAMEIIKKFKKDLAAMLRIINARKERKRRGADTSIGIIGLLLTTA 120

Senegal/1984 ····················································V······· 120

Malaysia/1966 ········································K········V··V······· 120

Cambodia/2010 ········································K····T···V··V······· 120

Puerto_Rico/2015 ···················T····················K········V··V······· 120

**pre M**

**Capsid**

Uganda/1947 MAAEITRRGSAYYMYLDRSDAGKAISFATTLGVNKCHVQIMDLGHMCDATMSYECPMLDE 180

Senegal/1984 ···························································· 180

Malaysia/1966 ··V·V······················P········YI······················ 180

Cambodia/2010 ··V·V····N············E····P··M·M···YI······················ 180

Puerto_Rico/2015 ····V·············N···E····P····M···YI······················ 180

Uganda/1947 GVEPDDVDCWCNTTSTWVVYGTCHHKKGEARRSRRAVTLPSHSTRKLQTRSQTWLESREY 240

Senegal/1984 ···························································· 240

Malaysia/1966 ···························································· 240

Cambodia/2010 ···························································· 240

Puerto_Rico/2015 ···························································· 240

**E protein**

**pre M**

Uganda/1947 TKHLIKVENWIFRNPGFALVAVAIAWLLGSSTSQKVIYLVMILLIAPAYSIRCIGVSNRD 300

Senegal/1984 ···························································· 300

Malaysia/1966 ·····R·············A·A······································ 300

Cambodia/2010 ·····R·············A·A······································ 300

Puerto_Rico/2015 ·····R·············A·A······································ 300

Uganda/1947 FVEGMSGGTWVDVVLEHGGCVTVMAQDKPTVDIELVTTTVSNMAEVRSYCYEASISDMAS 360

Senegal/1984 ···························································· 360

Malaysia/1966 ···························································· 360

Cambodia/2010 ···························································· 360

Puerto_Rico/2015 ···························································· 360

Uganda/1947 DSRCPTQGEAYLDKQSDTQYVCKRTLVDRGWGNGCGLFGKGSLVTCAKFTCSKKMTGKSI 420

Senegal/1984 ···························································· 420

Malaysia/1966 ·················································A·········· 420

Cambodia/2010 ·················································A·········· 420

Puerto_Rico/2015 ·················································A·········· 420

Uganda/1947 QPENLEYRIMLSVHGSQHSGMIVNDTGYETDENRAKVEVTPNSPRAEATLGGFGSLGLDC 480

Senegal/1984 ···························H································ 480

Malaysia/1966 ·························I·H··········I····················· 480

Cambodia/2010 ···························H··········I····················· 480

Puerto_Rico/2015 ···························H··········I····················· 480

Uganda/1947 EPRTGLDFSDLYYLTMNNKHWLVHKEWFHDIPLPWHAGADTGTPHWNNKEALVEFKDAHA 540

Senegal/1984 ···························································· 540

Malaysia/1966 ···························································· 540

Cambodia/2010 ···························································· 540

Puerto_Rico/2015 ···························································· 540

Uganda/1947 KRQTVVVLGSQEGAVHTALAGALEAEMDGAKGKLFSGHLKCRLKMDKLRLKGVSYSLCTA 600

Senegal/1984 ································R··························· 600

Malaysia/1966 ································R·S························· 600

Cambodia/2010 ································R·S························· 600

Puerto_Rico/2015 ································R·S························· 600

Uganda/1947 AFTFTKVPAETLHGTVTVEVQYAGTDGPCKIPVQMAVDMQTLTPVGRLITANPVITESTE 660

Senegal/1984 ······························V·A··························· 660

Malaysia/1966 ······I·······················V·A··························· 660

Cambodia/2010 ······I·······················V·A··························· 660

Puerto_Rico/2015 ······I·······················V·A··························· 660

Uganda/1947 NSKMMLELDPPFGDSYIVIGVGDKKITHHWHRSGSTIGKAFEATVRGAKRMAVLGDTAWD 720

Senegal/1984 ···························································· 720

Malaysia/1966 ···························································· 720

Cambodia/2010 ······················E····································· 720

Puerto_Rico/2015 ······················E····································· 720

Uganda/1947 FGSVGGVFNSLGKGIHQIFGAAFKSLFGGMSWFSQILIGTLLVWLGLNTKNGSISLTCLA 780

Senegal/1984 ···························································· 780

Malaysia/1966 ······AL···················································· 780

Cambodia/2010 ······AL················································M··· 780

Puerto_Rico/2015 ······AL··································M·············M··· 780

**NS1**

**E protein**

Uganda/1947 LGGVMIFLSTAVSADVGCSVDFSKKETRCGTGVFIYNDVEAWRDRYKYHPDSPRRLAAAV 840

Senegal/1984 ··································V························· 840

Malaysia/1966 ····L·····························V························· 840

Cambodia/2010 ····L·····························V························· 840

Puerto_Rico/2015 ····L·····························V························· 840

Uganda/1947 KQAWEEGICGISSVSRMENIMWKSVEGELNAILEENGVQLTVVVGSVKNPMWRGPQRLPV 900

Senegal/1984 ···························································· 900

Malaysia/1966 ·····D················R····································· 900

Cambodia/2010 ·····D················R····································· 900

Puerto_Rico/2015 ·····D················R····································· 900

Uganda/1947 PVNELPHGWKAWGKSYFVRAAKTNNSFVVDGDTLKECPLEHRAWNSFLVEDHGFGVFHTS 960

Senegal/1984 ·······································K···············I···· 960

Malaysia/1966 ·······································K···················· 960

Cambodia/2010 ·······································K···················· 960

Puerto_Rico/2015 ·······································K···················· 960

Uganda/1947 VWLKVREDYSLECDPAVIGTAVKGREAAHSDLGYWIESEKNDTWRLKRAHLIEMKTCEWP 1020

Senegal/1984 ························K·····················R············· 1020

Malaysia/1966 ························K··V································ 1020

Cambodia/2010 ·····················A··K··V································ 1020

Puerto_Rico/2015 ························K··V································ 1020

Uganda/1947 KSHTLWTDGVEESDLIIPKSLAGPLSHHNTREGYRTQVKGPWHSEELEIRFEECPGTKVY 1080

Senegal/1984 ·····································L·····················H 1080

Malaysia/1966 ·········I·················································H 1080

Cambodia/2010 ·········I···························M·····················H 1080

Puerto_Rico/2015 ·········I···························M·····················H 1080

Uganda/1947 VEETCGTRGPSLRSTTASGRVIEEWCCRECTMPPLSFRAKDGCWYGMEIRPRKEPESNLV 1140

Senegal/1984 ···························································· 1140

Malaysia/1966 ···························································· 1140

Cambodia/2010 ···························································· 1140

Puerto_Rico/2015 ···························································· 1140

**NS1**

**NS2A**

Uganda/1947 RSMVTAGSTDHMDHFSLGVLVILLMVQEGLKKRMTTKIIMSTSMAVLVVMILGGFSMSDL 1200

Senegal/1984 ················································A·V········· 1200

Malaysia/1966 ·······································I········A··········· 1200

Cambodia/2010 ·······································I········A··········· 1200

Puerto_Rico/2015 ·······································I········A··········· 1200

Uganda/1947 AKLVILMGATFAEMNTGGDVAHLALVAAFKVRPALLVSFIFRANWTPRESMLLALASCLL 1260

Senegal/1984 ···························································· 1260

Malaysia/1966 ···A·····················I·································· 1260

Cambodia/2010 ···A·····················I·································· 1260

Puerto_Rico/2015 ···A·····················I·································· 1260

Uganda/1947 QTAISALEGDLMVLINGFALAWLAIRAMAVPRTDNIALPILAALTPLARGTLLVAWRAGL 1320

Senegal/1984 ·········E····V·······················A····················· 1320

Malaysia/1966 ··V·································T·A····················· 1320

Cambodia/2010 ·············P··············V·······T·A····················· 1320

Puerto_Rico/2015 ····························V·······T·A····················· 1320

**NS2B**

**NS2A**

Uganda/1947 ATCGGIMLLSLKGKGSVKKNLPFVMALGLTAVRVVDPINVVGLLLLTRSGKRSWPPSEVL 1380

Senegal/1984 ·····F···························I·························· 1380

Malaysia/1966 ·····F···························L·························· 1380

Cambodia/2010 ·····F···························L·························· 1380

Puerto_Rico/2015 ·····F···························L·························· 1380

Uganda/1947 TAVGLICALAGGFAKADIEMAGPMAAVGLLIVSYVVSGKSVDMYIERAGDITWEKDAEVT 1440

Senegal/1984 ···························································· 1440

Malaysia/1966 ···························································· 1440

Cambodia/2010 ···························································· 1440

Puerto_Rico/2015 ···························································· 1440

Uganda/1947 GNSPRLDVALDESGDFSLVEEDGPPMREIILKVVLMAICGMNPIAIPFAAGAWYVYVKTG 1500

Senegal/1984 ···························································· 1500

Malaysia/1966 ····················D···············T······················· 1500

Cambodia/2010 ····················D······································· 1500

Puerto_Rico/2015 ····················D···············T······················· 1500

**NS3**

**NS2B**

Uganda/1947 KRSGALWDVPAPKEVKKGETTDGVYRVMTRRLLGSTQVGVGVMQEGVFHTMWHVTKGAAL 1560

Senegal/1984 ···························································· 1560

Malaysia/1966 ·························································S·· 1560

Cambodia/2010 ·························································S·· 1560

Puerto_Rico/2015 ·························································S·· 1560

Uganda/1947 RSGEGRLDPYWGDVKQDLVSYCGPWKLDAAWDGLSEVQLLAVPPGERARNIQTLPGIFKT 1620

Senegal/1984 ···························································· 1620

Malaysia/1966 ·································H·························· 1620

Cambodia/2010 ·································H·························· 1620

Puerto_Rico/2015 ·································H·························· 1620

Uganda/1947 KDGDIGAVALDYPAGTSGSPILDKCGRVIGLYGNGVVIKNGSYVSAITQGKREEETPVEC 1680

Senegal/1984 ·······················································A···· 1680

Malaysia/1966 ··················································R········· 1680

Cambodia/2010 ··················································R········· 1680

Puerto_Rico/2015 ··················································R········· 1680

Uganda/1947 FEPSMLKKKQLTVLDLHPGAGKTRRVLPEIVREAIKKRLRTVILAPTRVVAAEMEEALRG 1740

Senegal/1984 ······R····················································· 1740

Malaysia/1966 ····································T······················· 1740

Cambodia/2010 ····································T······················· 1740

Puerto_Rico/2015 ····································T······················· 1740

Uganda/1947 LPVRYMTTAVNVTHSGTEIVDLMCHATFTSRLLQPIRVPNYNLYIMDEAHFTDPSSIAAR 1800

Senegal/1984 ···························································· 1800

Malaysia/1966 ···························································· 1800

Cambodia/2010 ···························································· 1800

Puerto_Rico/2015 ···························································· 1800

Uganda/1947 GYISTRVEMGEAAAIFMTATPPGTRDAFPDSNSPIMDTEVEVPERAWSSGFDWVTDHSGK 1860

Senegal/1984 ···························································· 1860

Malaysia/1966 ···························································· 1860

Cambodia/2010 ···························································· 1860

Puerto_Rico/2015 ···························································· 1860

Uganda/1947 TVWFVPSVRNGNEIAACLTKAGKRVIQLSRKTFETEFQKTKNQEWDFVITTDISEMGANF 1920

Senegal/1984 ·I·························································· 1920

Malaysia/1966 ················································V··········· 1920

Cambodia/2010 ·········································H······V··········· 1920

Puerto_Rico/2015 ·········································H······V··········· 1920

Uganda/1947 KADRVIDSRRCLKPVILDGERVILAGPMPVTHASAAQRRGRIGRNPNKPGDEYMYGGGCA 1980

Senegal/1984 ·········································V·················· 1980

Malaysia/1966 ···························································· 1980

Cambodia/2010 ·····················································L······ 1980

Puerto_Rico/2015 ·····················································L······ 1980

Uganda/1947 ETDEGHAHWLEARMLLDNIYLQDGLIASLYRPEADKVAAIEGEFKLRTEQRKTFVELMKR 2040

Senegal/1984 ····D······················································· 2040

Malaysia/1966 ····D······················································· 2040

Cambodia/2010 ····D······················································· 2040

Puerto_Rico/2015 ····D······················································· 2040

Uganda/1947 GDLPVWLAYQVASAGITYTDRRWCFDGTTNNTIMEDSVPAEVWTKYGEKRVLKPRWMDAR 2100

Senegal/1984 ···························································· 2100

Malaysia/1966 ············································R··············· 2100

Cambodia/2010 ············································R··············· 2100

Puerto_Rico/2015 ············································RH·············· 2100

**NS4A**

**NS3**

Uganda/1947 VCSDHAALKSFKEFAAGKRGAALGVMEALGTLPGHMTERFQEAIDNLAVLMRAETGSRPY 2160

Senegal/1984 ··························D································· 2160

Malaysia/1966 ······················F····································· 2160

Cambodia/2010 ······················F····································· 2160

Puerto_Rico/2015 ······················F····································· 2160

Uganda/1947 KAAAAQLPETLETIMLLGLLGTVSLGIFFVLMRNKGIGKMGFGMVTLGASAWLMWLSEIE 2220

Senegal/1984 ···························································· 2220

Malaysia/1966 ···························································· 2220

Cambodia/2010 ···························································· 2220

Puerto_Rico/2015 ···························································· 2220

**NS4B**

**protein 2K**

**NS4A**

Uganda/1947 PARIACVLIVVFLLLVVLIPEPEKQRSPQDNQMAIIIMVAVGLLGLITANELGWLERTKN 2280

Senegal/1984 ···························································S 2280

Malaysia/1966 ···························································S 2280

Cambodia/2010 ···························································S 2280

Puerto_Rico/2015 ···························································S 2280

Uganda/1947 DIAHLMGRREEGATMGFSMDIDLRPASAWAIYAALTTLITPAVQHAVTTSYNNYSLMAMA 2340

Senegal/1984 ········K···T··············································· 2340

Malaysia/1966 ·LG························································· 2340

Cambodia/2010 ·LS···········I······················F······················ 2340

Puerto_Rico/2015 ·LS···········I······················F······················ 2340

Uganda/1947 TQAGVLFGMGKGMPFYAWDLGVPLLMMGCYSQLTPLTLIVAIILLVAHYMYLIPGLQAAA 2400

Senegal/1984 ···················F········································ 2400

Malaysia/1966 ···················F········································ 2400

Cambodia/2010 ···················F······I································· 2400

Puerto_Rico/2015 ···················F······I································· 2400

Uganda/1947 ARAAQKRTAAGIMKNPVVDGIVVTDIDTMTIDPQVEKKMGQVLLIAVAISSAVLLRTAWG 2460

Senegal/1984 ················································V··········· 2460

Malaysia/1966 ···························································· 2460

Cambodia/2010 ················································V···I·S····· 2460

Puerto_Rico/2015 ················································V···I·S····· 2460

**NS4B**

Uganda/1947 WGEAGALITAATSTLWEGSPNKYWNSSTATSLCNIFRGSYLAGASLIYTVTRNAGLVKRR 2520

Senegal/1984 ···························································· 2520

Malaysia/1966 ···························································· 2520

Cambodia/2010 ···························································· 2520

Puerto_Rico/2015 ···························································· 2520

**NS5**

Uganda/1947 GGGTGETLGEKWKARLNQMSALEFYSYKKSGITEVCREEARRALKDGVATGGHAVSRGSA 2580

Senegal/1984 ···························································· 2580

Malaysia/1966 ···························································· 2580

Cambodia/2010 ···························································· 2580

Puerto_Rico/2015 ···························································· 2580

Uganda/1947 KLRWLVERGYLQPYGKVVDLGCGRGGWSYYAATIRKVQEVRGYTKGGPGHEEPMLVQSYG 2640

Senegal/1984 ·············H·············································· 2640

Malaysia/1966 ·················I······················K············T······ 2640

Cambodia/2010 ·················I······················K··················· 2640

Puerto_Rico/2015 ·················I············V·········K············V······ 2640

Uganda/1947 WNIVRLKSGVDVFHMAAEPCDTLLCDIGESSSSPEVEETRTLRVLSMVGDWLEKRPGAFC 2700

Senegal/1984 ···························································· 2700

Malaysia/1966 ··················S···················A····················· 2700

Cambodia/2010 ······································A····················· 2700

Puerto_Rico/2015 ······································A····················· 2700

Uganda/1947 IKVLCPYTSTMMETMERLQRRHGGGLVRVPLSRNSTHEMYWVSGAKSNIIKSVSTTSQLL 2760

Senegal/1984 ···························································· 2760

Malaysia/1966 ··············L······Y······································ 2760

Cambodia/2010 ··············L······Y··························T··········· 2760

Puerto_Rico/2015 ··············L······Y··························T··········· 2760

Uganda/1947 LGRMDGPRRPVKYEEDVNLGSGTRAVASCAEAPNMKIIGRRIERIRNEHAETWFLDENHP 2820

Senegal/1984 ····E·················································F····· 2820

Malaysia/1966 ··································L····N·V····S·······F····· 2820

Cambodia/2010 ··························V············N······S·······F····· 2820

Puerto_Rico/2015 ··························V············N······S·······F····· 2820

Uganda/1947 YRTWAYHGSYEAPTQGSASSLVNGVVRLLSKPWDVVTGVTGIAMTDTTPYGQQRVFKEKV 2880

Senegal/1984 ···························································· 2880

Malaysia/1966 ·····················I······································ 2880

Cambodia/2010 ·····················I······································ 2880

Puerto_Rico/2015 ·····················I······································ 2880

Uganda/1947 DTRVPDPQEGTRQVMNIVSSWLWKELGKRKRPRVCTKEEFINKVRSNAALGAIFEEEKEW 2940

Senegal/1984 ················M··········································· 2940

Malaysia/1966 ················M···········H······························· 2940

Cambodia/2010 ···············SM···········H······························· 2940

Puerto_Rico/2015 ···············SM···········H······························· 2940

Uganda/1947 KTAVEAVNDPRFWALVDREREHHLRGECHSCVYNMMGKREKKQGEFGKAKGSRAIWYMWL 3000

Senegal/1984 ·················K·········································· 3000

Malaysia/1966 ·················K··········Q······························· 3000

Cambodia/2010 ·················K··········Q······························· 3000

Puerto_Rico/2015 ·················K··········Q······························· 3000

Uganda/1947 GARFLEFEALGFLNEDHWMGRENSGGGVEGLGLQRLGYILEEMNRAPGGKMYADDTAGWD 3060

Senegal/1984 ···························································· 3060

Malaysia/1966 ······································V····S·T·············· 3060

Cambodia/2010 ······································V····S·I···R·········· 3060

Puerto_Rico/2015 ······································V····S·I···R·········· 3060

Uganda/1947 TRISKFDLENEALITNQMEEGHRTLALAVIKYTYQNKVVKVLRPAEGGKTVMDIISRQDQ 3120

Senegal/1984 ···························································· 3120

Malaysia/1966 ····R··············K···A····I·················R············· 3120

Cambodia/2010 ····R··············K···A····I·················K············· 3120

Puerto_Rico/2015 ····R··············K···A····I·················K············· 3120

Uganda/1947 RGSGQVVTYALNTFTNLVVQLIRNMEAEEVLEMQDLWLLRKPEKVTRWLQSNGWDRLKRM 3180

Senegal/1984 ·································H·························· 3180

Malaysia/1966 ········································R·····S············· 3180

Cambodia/2010 ········································RS····N············· 3180

Puerto_Rico/2015 ········································RS····N············· 3180

Uganda/1947 AVSGDDCVVKPIDDRFAHALRFLNDMGKVRKDTQEWKPSTGWSNWEEVPFCSHHFNKLYL 3240

Senegal/1984 ··························································H· 3240

Malaysia/1966 ····················································Y······· 3240

Cambodia/2010 ··········································D···············H· 3240

Puerto_Rico/2015 ··········································D···············H· 3240

Uganda/1947 KDGRSIVVPCRHQDELIGRARVSPGAGWSIRETACLAKSYAQMWQLLYFHRRDLRLMANA 3300

Senegal/1984 ···························································· 3300

Malaysia/1966 ···························································· 3300

Cambodia/2010 ···························································· 3300

Puerto_Rico/2015 ···························································· 3300

Uganda/1947 ICSAVPVDWVPTGRTTWSIHGKGEWMTTEDMLMVWNRVWIEENDHMEDKTPVTKWTDIPY 3360

Senegal/1984 ···························································· 3360

Malaysia/1966 ···S····························V··························· 3360

Cambodia/2010 ···S····························V··························· 3360

Puerto_Rico/2015 ···S····························V··························· 3360

·························································································

Uganda/1947 LGKREDLWCGSLIGHRPRTTWAENIKDTVNMVRRIIGDEEKYMDYLSTQVRYLGEEGSTP 3420

Senegal/1984 ···························································· 3420

Malaysia/1966 ···························································· 3420

Cambodia/2010 ··························N····M··········V················· 3420

Puerto_Rico/2015 ··························N································· 3420

Uganda/1947 GVL 3423

Senegal/1984 ··· 3423

Malaysia/1966 ··· 3423

Cambodia/2010 ··· 3423

Puerto_Rico/2015 ··· 3423
