# Supplementary material for: A novel Zika virus mouse model reveals strain specific differences in virus pathogenesis and host inflammatory immune responses
Source: PLoS Pathog. 2017 Mar 9;13(3):e1006258. doi: 10.1371/journal.ppat.1006258 (PMC5373643; doi:10.1371/journal.ppat.1006258)
Supplement: S2 Table — (DOCX) [file ppat.1006258.s006.docx]

| name | Genbank acc.# | name | Genbank acc.# |
| --- | --- | --- | --- |
| Mexico/2016 | KX446951 | Haiti/2014 | KU509998 |
| Mexico/2015 | KX247632 | Haiti/2016 | KX051563 |
| Honduras/2016 | KX262887 | French Polynesia/2013 | KX369547 |
| Honduras/2015 | KX694534 | Tonga/2016 | KX806557 |
| Guatemala/2015 | KU501217 | Samoa/2016 | KX185891 |
| Guatemala/2016 | KU870645 | French Polynesia/2014 | KX447517 |
| Brazil/2016 | KU926309 | Singapore/2016 | KX813683 |
| Dominican Republic/2016 | KU853012 | Thailand/2014 | KU681081 |
| USA/2016 | KX842449 | Thailand/2013 | KX694532 |
| Guadeloupe/2016 | KX673530 | Cambodia/2010 | KU955593 |
| Ecuador/2016 | KX879603 | Philippines/2012 | KU681082 |
| French Guiana/2015 | KU758877 | Micronesia/2007 | EU545988 |
| Suriname/2016 | KU937936 | Malaysia/1966 | KX377336 |
| Puerto Rico/2015 | KX377337??? | Central African Republic/1979 | KF268950 |
| China/2016 | KU761564 | Central African Republic/1976 | KF268948 |
| Suriname/2015 | KU312312 | Central African Republic/1980 | KF268949 |
| Colombia/2016 | KX247646 | Central African Republic/1968 | KF383115 |
| Venezuela/2016 | KX702400 | Senegal/2001 | KF383119 |
| Panama/2015 | KX156775 | Uganda/1947 | KU955594 |
| Panama/2016 | KX198135 | Senegal/1984 | HQ234501.1 |
| Colombia/2015 | KX548902 | Senegal/1968 | KF383116 |
| Martinique/2015 | KU647676 | Senegal/1997 | KF383117 |
| Brazil/2015 | KU321639 | Nigeria/1968 | KU963574 |
